# 

**Published:** 1868-04

**Authors:** 


### CONTENTS.

| | PAGE. |
|---|---|
| Medical Cliniques, by A. M. Carpenter, M. D | 29 |
| Surgical Cliniques, by Prof. J. C. Hughes, M. D | 31 |
| Medicine not an Exact Science, by J. W. H. Baker, M. D | 37 |
| Misplaced Ovary, by Edward Whinery, M. D | 46 |
| Report of Four Successful Operations, upon the Same Individual, for Stone in the Bladder, by Prof. J. C. Hughes, M. D | 47 |
| Asiatic Cholera, by Philip Harvey, M. D | 52 |
| Report on Criminal Abortion, by J. C. Stone, M. D | 59 |
| Hypodermic Injections, by A. S. Maxwell, M. D | 66 |
| Anæsthesia, by A. T. Hudson, M. D | 70 |
| Lithotomy, (a novel case,) by Edward Whinery, M. D | 78 |
| Meteorology and Medical Topography, by S. D. Richardson, M. D | 79 |
| Report on New Remedies, by J. W. H. Baker, M. D | 80 |
| Editorial | 86-88 |
| Reviews | 88 |
| Advertisements | 89 |

